# Tris(methylammonium thiocyanurate) monohydrate

**DOI:** 10.1107/S1600536810050312

**Published:** 2010-12-08

**Authors:** Yimin Hou, Yunxia Yang

**Affiliations:** a Henan University of Traditional Chinese Medicine, Zhengzhou 450008, People’s Republic of China; b Key Laboratory of Polymer Materials of Gansu Province, Ministry of Education, College of Chemistry and Chemical Engineering, Northwest Normal University, Lanzhou 730070, Gansu, People’s Republic of China

## Abstract

In the title compound, 3[(CH_3_)_3_HN^+^]·3C_3_H_2_N_3_S_3_^−^·H_2_O, two independent trithio­cyanurate anions construct a planar hydrogen-bonded ribbon with two N—H⋯S hydrogen bonds linking each pair of adjacent anions in the chain. The third independent anion and the water mol­ecule form a chain by way of N—H⋯S and O—H⋯S contacts, which propagates parallel to the ribbon. The chains and ribbons are cross-linked by O—H⋯S hydrogen bonds, generating sheets. The three independent trimethyl­ammonium cations are contained between the sheets by way of various N—H⋯S and N—H⋯N contacts.

## Related literature

For hydrogen-bond formation in the compounds of trithio­cyanuric acid, see: Dean *et al.* (2004 ▶).
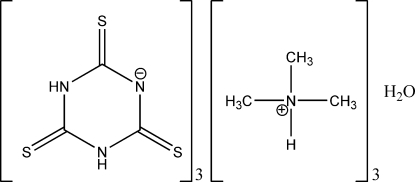


## Experimental

### 

#### Crystal data

3C_3_H_10_N^+^·3C_3_H_2_N_3_S_3_^−^·H_2_O
*M* *_r_* = 727.23
Triclinic, 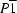

*a* = 11.3466 (1) Å
*b* = 12.6474 (1) Å
*c* = 12.8135 (1) Å
α = 76.950 (1)°
β = 84.762 (1)°
γ = 82.274 (1)°
*V* = 1771.51 (3) Å^3^
*Z* = 2
Mo *K*α radiation
μ = 0.60 mm^−1^
*T* = 296 K
0.55 × 0.40 × 0.09 mm

#### Data collection

Bruker APEXII CCD diffractometer
Absorption correction: multi-scan *SADABS* (Bruker, 2007 ▶) *T*_min_ = 0.735, *T*_max_ = 0.948
14356 measured reflections
6243 independent reflections
5406 reflections with *I* > 2σ(*I*)
*R*_int_ = 0.016

#### Refinement

*R*[*F* ^2^ > 2σ(*F* ^2^)] = 0.036
*wR*(*F* ^2^) = 0.103
*S* = 1.05
6243 reflections
388 parameters
12 restraints
H atoms treated by a mixture of independent and constrained refinement
Δρ_max_ = 0.43 e Å^−3^
Δρ_min_ = −0.28 e Å^−3^

### 

Data collection: *APEX2* (Bruker, 2007 ▶); cell refinement: *SAINT* (Bruker, 2007 ▶); data reduction: *SAINT*; program(s) used to solve structure: *SHELXS97* (Sheldrick, 2008 ▶); program(s) used to refine structure: *SHELXL97* (Sheldrick, 2008 ▶); molecular graphics: *SHELXTL* (Sheldrick, 2008 ▶); software used to prepare material for publication: *SHELXL97* and *publCIF* (Westrip, 2010 ▶).

## Supplementary Material

Crystal structure: contains datablocks I, global. DOI: 10.1107/S1600536810050312/hb5752sup1.cif

Structure factors: contains datablocks I. DOI: 10.1107/S1600536810050312/hb5752Isup2.hkl

Additional supplementary materials:  crystallographic information; 3D view; checkCIF report

## Figures and Tables

**Table 1:** Hydrogen-bond geometry (Å, °)

*D*—H⋯*A*	*D*—H	H⋯*A*	*D*⋯*A*	*D*—H⋯*A*
O1*W*—H1*WA*⋯S3	0.86	2.57	3.284 (2)	140
O1*W*—H1*WB*⋯S9^i^	0.87	2.52	3.355 (2)	161
N2—H2⋯S4^ii^	0.85 (1)	2.48 (1)	3.3196 (17)	168 (2)
N3—H3⋯S6^iii^	0.85 (1)	2.48 (1)	3.3203 (17)	168 (2)
N4—H4⋯S2^ii^	0.86 (1)	2.45 (1)	3.2962 (17)	169 (2)
N5—H5⋯S1^iii^	0.86 (1)	2.49 (1)	3.3316 (17)	167 (2)
N7—H7⋯O1*W*	0.86 (1)	2.03 (1)	2.878 (3)	172 (3)
N9—H9⋯S8^iv^	0.86 (1)	2.63 (1)	3.470 (2)	167 (3)
N10—H10⋯N6	0.87 (1)	1.95 (1)	2.804 (3)	168 (3)
N11—H11⋯N1	0.86 (1)	1.95 (1)	2.795 (2)	164 (3)
N12—H12⋯S7^v^	0.85 (1)	2.69 (2)	3.439 (2)	148 (3)

Symmetry codes: (i) 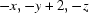; (ii) 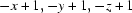; (iii) 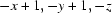; (iv) 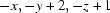; (v) 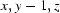.

**Fig. 1.**
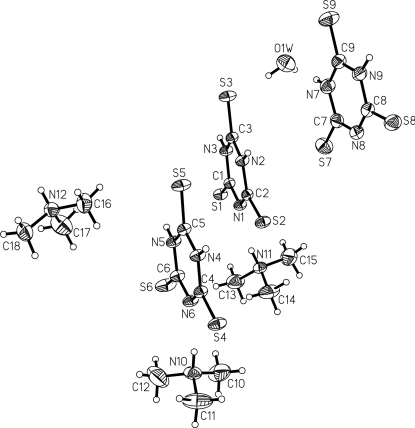
The title compound at the 30% probability level; hydrogen atoms are drawn as spheres of arbitrary radius.

**Fig. 2.**
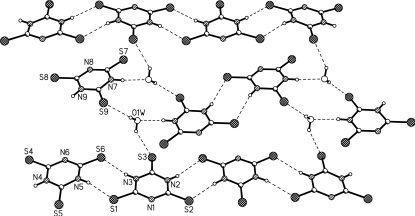
Hydrogen-bonded linking pattern of the separated unit in the crystal structure of the title compound.

**Fig. 3.**
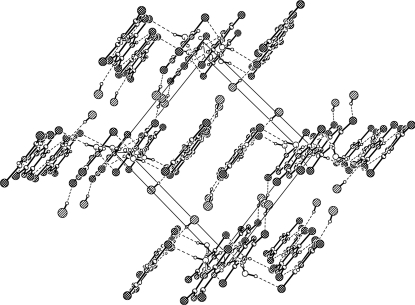
Packing diagram of the title compound; all hydrogen atoms bonded to carbon and carbon atoms of the trimethylammonium cations are omitted for clarity and the cations are represented with the hatched spheres.

## supplementary crystallographic information

### Comment

Trithiocyanuric acid, which can be regarded as the polymer of three thiourea molecules, have a strong tendency to form various hydrogen bonds (Dean *et al.* 2004). Here we report the crystal structure of the title ammonium water-trithiocyanurate, 3[(CH_3_)_3_HN^+^].3C_3_H_2_N_3_S_3_^-^.H_2_O, (I). In this structure, two independent trithiocyanurate anions containing S1 and S4 atoms firstly form the hydrogen-bonded ribbon parallel to (010) plane by four N—H···S hydrogen bonds, and then between two planar ribbons, there exist a totally different waving ribbon composed of the third trithiocyanurate anion and the only water molecule, in which the anion links the water molecule with varied N—H···S and O—H···S contacts (Fig. 2). With the existence of one independent O—H···S interaction between the aforementioned planar hydrogen-bonded ribbon and the waving ribbon, two planar ribbons and one waving ribbon can yield the larger hydrogen-bonded unit as shown in Fig. 3. The ammonium cations, with the existence of N—H···S and N—H···N interactions, are stably accommodated to the intervals of these separated units to form the stable crystal structure.

### Experimental

Trithiocyanuric acid (0.044 g, 0.25 mmol) was dissolved in a water-ethanol (1:2 *v*/*v*) mixture and a 33% solution of trimethyl amine was added to neutralize the acid. Colorless block crystals separated after several weeks.

### Refinement

Nitrogen-bound H-atoms were placed in calculated positions (N—H 0.86 Å) and were included in the refinement in the riding model approximation, with *U*(H) set to 1.5*U*(N). The water H-atom was similarly treated.

### Crystal data

**Table:**

3C_3_H_10_N^+^·3C_3_H_2_N_3_S_3_^−^·H_2_O	*Z* = 2
*M**_r_* = 727.23	*F*(000) = 764
Triclinic, *P*1	*D*_x_ = 1.363 Mg m^−^^3^
Hall symbol: -P 1	Mo *K*α radiation, λ = 0.71073 Å
*a* = 11.3466 (1) Å	Cell parameters from 8448 reflections
*b* = 12.6474 (1) Å	θ = 2.4–27.6°
*c* = 12.8135 (1) Å	µ = 0.60 mm^−^^1^
α = 76.950 (1)°	*T* = 296 K
β = 84.762 (1)°	Block, colorless
γ = 82.274 (1)°	0.55 × 0.40 × 0.09 mm
*V* = 1771.51 (3) Å^3^	

### Data collection

**Table:**

Bruker APEXII CCD diffractometer	6243 independent reflections
Radiation source: fine-focus sealed tube	5406 reflections with *I* > 2σ(*I*)
graphite	*R*_int_ = 0.016
phi and ω scans	θ_max_ = 25.0°, θ_min_ = 2.5°
Absorption correction: multi-scan *SADABS* (Bruker, 2007)	*h* = −13→13
*T*_min_ = 0.735, *T*_max_ = 0.948	*k* = −15→14
14356 measured reflections	*l* = −15→15

### Refinement

**Table:**

Refinement on *F*^2^	Primary atom site location: structure-invariant direct methods
Least-squares matrix: full	Secondary atom site location: difference Fourier map
*R*[*F*^2^ > 2σ(*F*^2^)] = 0.036	Hydrogen site location: inferred from neighbouring sites
*wR*(*F*^2^) = 0.103	H atoms treated by a mixture of independent and constrained refinement
*S* = 1.05	*w* = 1/[σ^2^(*F*_o_^2^) + (0.0524*P*)^2^ + 0.8093*P*] where *P* = (*F*_o_^2^ + 2*F*_c_^2^)/3
6243 reflections	(Δ/σ)_max_ = 0.002
388 parameters	Δρ_max_ = 0.43 e Å^−^^3^
12 restraints	Δρ_min_ = −0.28 e Å^−^^3^

### Special details

**Table:**

Geometry. All e.s.d.'s (except the e.s.d. in the dihedral angle between two l.s. planes) are estimated using the full covariance matrix. The cell e.s.d.'s are taken into account individually in the estimation of e.s.d.'s in distances, angles and torsion angles; correlations between e.s.d.'s in cell parameters are only used when they are defined by crystal symmetry. An approximate (isotropic) treatment of cell e.s.d.'s is used for estimating e.s.d.'s involving l.s. planes.
Refinement. Refinement of *F*^2^ against ALL reflections. The weighted *R*-factor *wR* and goodness of fit *S* are based on *F*^2^, conventional *R*-factors *R* are based on *F*, with *F* set to zero for negative *F*^2^. The threshold expression of *F*^2^ > σ(*F*^2^) is used only for calculating *R*-factors(gt) *etc*. and is not relevant to the choice of reflections for refinement. *R*-factors based on *F*^2^ are statistically about twice as large as those based on *F*, and *R*- factors based on ALL data will be even larger.

### Fractional atomic coordinates and isotropic or equivalent isotropic displacement parameters (Å^2^)

**Table:**

	*x*	*y*	*z*	*U*_iso_*/*U*_eq_	
S1	0.56389 (5)	0.64769 (5)	−0.12395 (4)	0.04362 (14)	
N1	0.55437 (14)	0.63614 (14)	0.08580 (12)	0.0364 (4)	
C1	0.49478 (16)	0.64115 (15)	−0.00121 (15)	0.0339 (4)	
O1W	0.0658 (2)	0.88067 (19)	−0.01243 (17)	0.0861 (6)	
H1WA	0.1088	0.8189	−0.0116	0.129*	
H1WB	0.0952	0.9280	−0.0651	0.129*	
S2	0.56375 (5)	0.62637 (5)	0.29317 (4)	0.04494 (15)	
N2	0.37391 (14)	0.62546 (14)	0.18974 (13)	0.0370 (4)	
H2	0.335 (2)	0.624 (2)	0.2500 (12)	0.055*	
C2	0.49461 (17)	0.62957 (15)	0.18250 (15)	0.0343 (4)	
S3	0.16216 (5)	0.63313 (6)	0.11585 (5)	0.05253 (16)	
N3	0.37320 (14)	0.64138 (14)	0.01016 (13)	0.0377 (4)	
H3	0.337 (2)	0.646 (2)	−0.0466 (13)	0.057*	
C3	0.30893 (17)	0.63316 (16)	0.10501 (15)	0.0364 (4)	
S4	0.80586 (5)	0.34932 (6)	0.59567 (4)	0.05156 (16)	
N4	0.61463 (14)	0.35390 (15)	0.49243 (13)	0.0407 (4)	
H4	0.577 (2)	0.361 (2)	0.5519 (13)	0.061*	
C4	0.73630 (17)	0.34937 (17)	0.48581 (16)	0.0381 (4)	
S5	0.40260 (5)	0.35735 (6)	0.42068 (5)	0.05557 (17)	
N5	0.61589 (15)	0.34088 (15)	0.31875 (13)	0.0404 (4)	
H5	0.581 (2)	0.343 (2)	0.2620 (14)	0.061*	
C5	0.54998 (17)	0.35067 (17)	0.41032 (16)	0.0380 (4)	
S6	0.80673 (5)	0.33438 (7)	0.18635 (5)	0.0681 (2)	
N6	0.79689 (14)	0.34433 (16)	0.39149 (13)	0.0428 (4)	
C6	0.73742 (18)	0.34035 (19)	0.30705 (16)	0.0424 (5)	
N10	1.04639 (17)	0.32035 (19)	0.3879 (2)	0.0643 (6)	
H10	0.9696 (10)	0.331 (3)	0.398 (3)	0.096*	
C10	1.0768 (4)	0.4212 (3)	0.3201 (4)	0.1236 (17)	
H10A	1.0568	0.4792	0.3580	0.185*	
H10B	1.0332	0.4372	0.2565	0.185*	
H10C	1.1607	0.4145	0.3004	0.185*	
N11	0.79117 (16)	0.68045 (18)	0.06442 (19)	0.0581 (5)	
H11	0.7233 (16)	0.655 (2)	0.078 (3)	0.087*	
C11	1.1012 (3)	0.2929 (4)	0.4905 (3)	0.1159 (16)	
H11A	1.0825	0.3528	0.5260	0.174*	
H11B	1.1860	0.2787	0.4786	0.174*	
H11C	1.0711	0.2289	0.5345	0.174*	
N12	0.50621 (19)	0.00292 (17)	0.28412 (18)	0.0569 (5)	
H12	0.4444 (18)	−0.015 (3)	0.262 (2)	0.085*	
C12	1.0776 (4)	0.2293 (4)	0.3325 (4)	0.1397 (19)	
H12A	1.0415	0.2473	0.2648	0.210*	
H12B	1.0489	0.1645	0.3759	0.210*	
H12C	1.1626	0.2169	0.3205	0.210*	
C13	0.8621 (3)	0.6173 (3)	−0.0050 (3)	0.0944 (11)	
H13A	0.8258	0.6298	−0.0722	0.142*	
H13B	0.8667	0.5410	0.0286	0.142*	
H13C	0.9409	0.6392	−0.0175	0.142*	
C14	0.8474 (3)	0.6615 (3)	0.1690 (3)	0.0917 (11)	
H14A	0.7998	0.7031	0.2150	0.137*	
H14B	0.9258	0.6841	0.1566	0.137*	
H14C	0.8527	0.5852	0.2026	0.137*	
C15	0.7683 (4)	0.7964 (3)	0.0190 (4)	0.1141 (15)	
H15A	0.7320	0.8074	−0.0480	0.171*	
H15B	0.8420	0.8280	0.0072	0.171*	
H15C	0.7156	0.8308	0.0678	0.171*	
C16	0.4718 (3)	0.0885 (2)	0.3457 (3)	0.0755 (8)	
H16A	0.4210	0.0612	0.4073	0.113*	
H16B	0.4300	0.1511	0.3012	0.113*	
H16C	0.5420	0.1090	0.3687	0.113*	
C17	0.5840 (3)	0.0438 (3)	0.1878 (3)	0.0974 (11)	
H17A	0.6051	−0.0125	0.1479	0.146*	
H17B	0.6551	0.0639	0.2098	0.146*	
H17C	0.5423	0.1066	0.1434	0.146*	
C18	0.5605 (3)	−0.0983 (2)	0.3525 (3)	0.0858 (10)	
H18A	0.5062	−0.1218	0.4127	0.129*	
H18B	0.6331	−0.0850	0.3776	0.129*	
H18C	0.5775	−0.1541	0.3116	0.129*	
N7	0.12163 (19)	0.92130 (18)	0.18856 (17)	0.0574 (5)	
H7	0.107 (3)	0.916 (3)	0.1258 (13)	0.086*	
S7	0.34408 (7)	0.92115 (7)	0.10863 (6)	0.0737 (2)	
C7	0.2383 (2)	0.92934 (19)	0.2065 (2)	0.0538 (6)	
S8	0.19623 (6)	0.96108 (6)	0.50398 (6)	0.06475 (19)	
N8	0.26245 (17)	0.94371 (16)	0.30300 (17)	0.0542 (5)	
C8	0.1747 (2)	0.95025 (18)	0.3790 (2)	0.0514 (5)	
S9	−0.11137 (7)	0.92925 (8)	0.23683 (7)	0.0800 (2)	
N9	0.05886 (18)	0.94998 (17)	0.35405 (17)	0.0529 (5)	
H9	0.0012 (19)	0.964 (2)	0.3986 (19)	0.079*	
C9	0.0290 (2)	0.9339 (2)	0.2601 (2)	0.0539 (6)	

### Atomic displacement parameters (Å^2^)

**Table:**

	*U*^11^	*U*^22^	*U*^33^	*U*^12^	*U*^13^	*U*^23^
S1	0.0367 (3)	0.0641 (3)	0.0296 (3)	−0.0079 (2)	0.0031 (2)	−0.0103 (2)
N1	0.0296 (8)	0.0493 (10)	0.0317 (8)	−0.0075 (7)	−0.0002 (6)	−0.0106 (7)
C1	0.0329 (9)	0.0372 (10)	0.0318 (10)	−0.0057 (8)	−0.0009 (8)	−0.0073 (8)
O1W	0.0859 (15)	0.0935 (15)	0.0732 (13)	0.0028 (12)	−0.0087 (11)	−0.0124 (11)
S2	0.0367 (3)	0.0688 (4)	0.0331 (3)	−0.0108 (2)	−0.0038 (2)	−0.0153 (2)
N2	0.0309 (8)	0.0528 (10)	0.0290 (8)	−0.0096 (7)	0.0027 (6)	−0.0114 (7)
C2	0.0327 (9)	0.0379 (10)	0.0334 (10)	−0.0064 (8)	−0.0011 (8)	−0.0091 (8)
S3	0.0302 (3)	0.0844 (4)	0.0445 (3)	−0.0137 (3)	−0.0001 (2)	−0.0138 (3)
N3	0.0314 (8)	0.0541 (10)	0.0287 (8)	−0.0080 (7)	−0.0024 (7)	−0.0095 (7)
C3	0.0330 (10)	0.0435 (11)	0.0339 (10)	−0.0084 (8)	−0.0005 (8)	−0.0092 (8)
S4	0.0354 (3)	0.0880 (4)	0.0377 (3)	−0.0090 (3)	−0.0046 (2)	−0.0251 (3)
N4	0.0304 (8)	0.0639 (11)	0.0310 (9)	−0.0094 (8)	0.0018 (7)	−0.0164 (8)
C4	0.0321 (10)	0.0489 (11)	0.0354 (10)	−0.0077 (8)	−0.0004 (8)	−0.0127 (9)
S5	0.0297 (3)	0.0942 (5)	0.0464 (3)	−0.0143 (3)	0.0000 (2)	−0.0197 (3)
N5	0.0323 (8)	0.0617 (11)	0.0308 (9)	−0.0107 (8)	−0.0010 (7)	−0.0150 (8)
C5	0.0337 (10)	0.0475 (11)	0.0344 (10)	−0.0096 (8)	−0.0011 (8)	−0.0102 (8)
S6	0.0400 (3)	0.1345 (7)	0.0371 (3)	−0.0152 (3)	0.0055 (2)	−0.0340 (4)
N6	0.0305 (8)	0.0669 (12)	0.0346 (9)	−0.0093 (8)	0.0005 (7)	−0.0176 (8)
C6	0.0351 (10)	0.0594 (13)	0.0351 (11)	−0.0085 (9)	0.0001 (8)	−0.0143 (9)
N10	0.0338 (10)	0.0679 (14)	0.0813 (15)	−0.0089 (10)	0.0012 (10)	0.0042 (11)
C10	0.101 (3)	0.090 (3)	0.164 (4)	−0.046 (2)	−0.049 (3)	0.041 (3)
N11	0.0320 (9)	0.0626 (13)	0.0851 (15)	−0.0111 (9)	0.0005 (10)	−0.0259 (11)
C11	0.0499 (17)	0.194 (4)	0.083 (2)	−0.014 (2)	0.0008 (16)	0.011 (3)
N12	0.0542 (12)	0.0575 (12)	0.0608 (13)	−0.0030 (10)	−0.0084 (10)	−0.0165 (10)
C12	0.128 (4)	0.116 (3)	0.172 (5)	0.047 (3)	−0.033 (3)	−0.048 (3)
C13	0.0535 (17)	0.131 (3)	0.116 (3)	−0.0040 (18)	−0.0003 (17)	−0.068 (2)
C14	0.0481 (16)	0.145 (3)	0.091 (2)	−0.0186 (18)	−0.0029 (15)	−0.040 (2)
C15	0.092 (3)	0.065 (2)	0.185 (4)	−0.0214 (18)	−0.031 (3)	−0.011 (2)
C16	0.086 (2)	0.0609 (16)	0.082 (2)	−0.0163 (15)	0.0168 (16)	−0.0243 (15)
C17	0.097 (3)	0.110 (3)	0.072 (2)	0.010 (2)	0.0189 (18)	−0.0126 (19)
C18	0.092 (2)	0.0576 (17)	0.106 (3)	0.0014 (15)	−0.032 (2)	−0.0100 (16)
N7	0.0571 (12)	0.0642 (13)	0.0535 (12)	−0.0103 (10)	−0.0052 (10)	−0.0158 (10)
S7	0.0663 (4)	0.0802 (5)	0.0686 (4)	−0.0057 (4)	0.0147 (3)	−0.0129 (4)
C7	0.0520 (13)	0.0428 (12)	0.0649 (15)	−0.0075 (10)	0.0010 (11)	−0.0089 (11)
S8	0.0578 (4)	0.0798 (5)	0.0621 (4)	−0.0077 (3)	−0.0098 (3)	−0.0245 (3)
N8	0.0469 (11)	0.0538 (11)	0.0646 (13)	−0.0095 (9)	−0.0011 (9)	−0.0174 (10)
C8	0.0485 (13)	0.0445 (12)	0.0630 (15)	−0.0089 (10)	−0.0031 (11)	−0.0136 (11)
S9	0.0556 (4)	0.1196 (7)	0.0727 (5)	−0.0239 (4)	−0.0104 (3)	−0.0271 (5)
N9	0.0463 (11)	0.0605 (12)	0.0567 (12)	−0.0117 (9)	−0.0006 (9)	−0.0200 (10)
C9	0.0527 (14)	0.0547 (14)	0.0558 (14)	−0.0122 (11)	−0.0021 (11)	−0.0122 (11)

### Geometric parameters (Å, °)

**Table:**

S1—C1	1.6819 (19)	C11—H11C	0.9600
N1—C1	1.341 (2)	N12—C16	1.470 (3)
N1—C2	1.348 (2)	N12—C18	1.471 (4)
C1—N3	1.374 (2)	N12—C17	1.486 (4)
O1W—H1WA	0.8619	N12—H12	0.851 (10)
O1W—H1WB	0.8691	C12—H12A	0.9600
S2—C2	1.6723 (19)	C12—H12B	0.9600
N2—C3	1.346 (3)	C12—H12C	0.9600
N2—C2	1.371 (2)	C13—H13A	0.9600
N2—H2	0.850 (10)	C13—H13B	0.9600
S3—C3	1.6585 (19)	C13—H13C	0.9600
N3—C3	1.349 (2)	C14—H14A	0.9600
N3—H3	0.854 (10)	C14—H14B	0.9600
S4—C4	1.675 (2)	C14—H14C	0.9600
N4—C5	1.347 (3)	C15—H15A	0.9600
N4—C4	1.370 (2)	C15—H15B	0.9600
N4—H4	0.857 (10)	C15—H15C	0.9600
C4—N6	1.345 (3)	C16—H16A	0.9600
S5—C5	1.658 (2)	C16—H16B	0.9600
N5—C5	1.354 (3)	C16—H16C	0.9600
N5—C6	1.373 (3)	C17—H17A	0.9600
N5—H5	0.855 (10)	C17—H17B	0.9600
S6—C6	1.682 (2)	C17—H17C	0.9600
N6—C6	1.340 (3)	C18—H18A	0.9600
N10—C10	1.436 (4)	C18—H18B	0.9600
N10—C11	1.456 (4)	C18—H18C	0.9600
N10—C12	1.473 (5)	N7—C9	1.347 (3)
N10—H10	0.865 (10)	N7—C7	1.385 (3)
C10—H10A	0.9600	N7—H7	0.856 (10)
C10—H10B	0.9600	S7—C7	1.666 (3)
C10—H10C	0.9600	C7—N8	1.346 (3)
N11—C13	1.447 (4)	S8—C8	1.680 (3)
N11—C15	1.448 (4)	N8—C8	1.336 (3)
N11—C14	1.493 (4)	C8—N9	1.381 (3)
N11—H11	0.863 (10)	S9—C9	1.658 (3)
C11—H11A	0.9600	N9—C9	1.346 (3)
C11—H11B	0.9600	N9—H9	0.857 (10)
			
C1—N1—C2	119.69 (16)	C18—N12—H12	105 (2)
N1—C1—N3	118.93 (16)	C17—N12—H12	107 (2)
N1—C1—S1	122.20 (14)	N10—C12—H12A	109.5
N3—C1—S1	118.87 (14)	N10—C12—H12B	109.5
H1WA—O1W—H1WB	106.8	H12A—C12—H12B	109.5
C3—N2—C2	124.13 (16)	N10—C12—H12C	109.5
C3—N2—H2	116.5 (17)	H12A—C12—H12C	109.5
C2—N2—H2	119.1 (17)	H12B—C12—H12C	109.5
N1—C2—N2	118.82 (17)	N11—C13—H13A	109.5
N1—C2—S2	121.75 (14)	N11—C13—H13B	109.5
N2—C2—S2	119.43 (14)	H13A—C13—H13B	109.5
C3—N3—C1	124.00 (16)	N11—C13—H13C	109.5
C3—N3—H3	118.8 (18)	H13A—C13—H13C	109.5
C1—N3—H3	117.2 (18)	H13B—C13—H13C	109.5
N2—C3—N3	114.34 (17)	N11—C14—H14A	109.5
N2—C3—S3	122.95 (14)	N11—C14—H14B	109.5
N3—C3—S3	122.71 (15)	H14A—C14—H14B	109.5
C5—N4—C4	124.14 (17)	N11—C14—H14C	109.5
C5—N4—H4	117.5 (18)	H14A—C14—H14C	109.5
C4—N4—H4	118.4 (18)	H14B—C14—H14C	109.5
N6—C4—N4	119.15 (17)	N11—C15—H15A	109.5
N6—C4—S4	121.71 (15)	N11—C15—H15B	109.5
N4—C4—S4	119.14 (14)	H15A—C15—H15B	109.5
C5—N5—C6	123.76 (17)	N11—C15—H15C	109.5
C5—N5—H5	119.5 (18)	H15A—C15—H15C	109.5
C6—N5—H5	116.3 (18)	H15B—C15—H15C	109.5
N4—C5—N5	114.15 (17)	N12—C16—H16A	109.5
N4—C5—S5	122.96 (15)	N12—C16—H16B	109.5
N5—C5—S5	122.90 (15)	H16A—C16—H16B	109.5
C6—N6—C4	119.38 (17)	N12—C16—H16C	109.5
N6—C6—N5	119.31 (17)	H16A—C16—H16C	109.5
N6—C6—S6	122.21 (15)	H16B—C16—H16C	109.5
N5—C6—S6	118.48 (15)	N12—C17—H17A	109.5
C10—N10—C11	113.8 (3)	N12—C17—H17B	109.5
C10—N10—C12	110.8 (4)	H17A—C17—H17B	109.5
C11—N10—C12	109.4 (3)	N12—C17—H17C	109.5
C10—N10—H10	104 (2)	H17A—C17—H17C	109.5
C11—N10—H10	110 (2)	H17B—C17—H17C	109.5
C12—N10—H10	108 (2)	N12—C18—H18A	109.5
N10—C10—H10A	109.5	N12—C18—H18B	109.5
N10—C10—H10B	109.5	H18A—C18—H18B	109.5
H10A—C10—H10B	109.5	N12—C18—H18C	109.5
N10—C10—H10C	109.5	H18A—C18—H18C	109.5
H10A—C10—H10C	109.5	H18B—C18—H18C	109.5
H10B—C10—H10C	109.5	C9—N7—C7	123.6 (2)
C13—N11—C15	115.1 (3)	C9—N7—H7	118 (2)
C13—N11—C14	109.7 (2)	C7—N7—H7	118 (2)
C15—N11—C14	110.3 (3)	N8—C7—N7	118.7 (2)
C13—N11—H11	107 (2)	N8—C7—S7	122.06 (19)
C15—N11—H11	108 (2)	N7—C7—S7	119.2 (2)
C14—N11—H11	107 (2)	C8—N8—C7	120.0 (2)
N10—C11—H11A	109.5	N8—C8—N9	118.8 (2)
N10—C11—H11B	109.5	N8—C8—S8	123.78 (19)
H11A—C11—H11B	109.5	N9—C8—S8	117.46 (18)
N10—C11—H11C	109.5	C9—N9—C8	123.9 (2)
H11A—C11—H11C	109.5	C9—N9—H9	116 (2)
H11B—C11—H11C	109.5	C8—N9—H9	119 (2)
C16—N12—C18	111.1 (2)	N9—C9—N7	114.7 (2)
C16—N12—C17	110.3 (2)	N9—C9—S9	121.64 (19)
C18—N12—C17	112.8 (3)	N7—C9—S9	123.69 (19)
C16—N12—H12	110 (2)		
			
C2—N1—C1—N3	1.1 (3)	N4—C4—N6—C6	−2.1 (3)
C2—N1—C1—S1	−179.13 (15)	S4—C4—N6—C6	177.44 (17)
C1—N1—C2—N2	1.6 (3)	C4—N6—C6—N5	−0.4 (3)
C1—N1—C2—S2	−178.39 (15)	C4—N6—C6—S6	179.78 (17)
C3—N2—C2—N1	−3.5 (3)	C5—N5—C6—N6	3.4 (3)
C3—N2—C2—S2	176.45 (16)	C5—N5—C6—S6	−176.76 (17)
N1—C1—N3—C3	−2.3 (3)	C9—N7—C7—N8	4.3 (4)
S1—C1—N3—C3	177.94 (16)	C9—N7—C7—S7	−175.60 (19)
C2—N2—C3—N3	2.4 (3)	N7—C7—N8—C8	−0.2 (3)
C2—N2—C3—S3	−177.34 (16)	S7—C7—N8—C8	179.74 (18)
C1—N3—C3—N2	0.6 (3)	C7—N8—C8—N9	−4.6 (3)
C1—N3—C3—S3	−179.71 (15)	C7—N8—C8—S8	176.47 (18)
C5—N4—C4—N6	1.9 (3)	N8—C8—N9—C9	6.1 (4)
C5—N4—C4—S4	−177.66 (17)	S8—C8—N9—C9	−174.96 (19)
C4—N4—C5—N5	0.9 (3)	C8—N9—C9—N7	−2.2 (3)
C4—N4—C5—S5	−179.48 (17)	C8—N9—C9—S9	177.35 (18)
C6—N5—C5—N4	−3.5 (3)	C7—N7—C9—N9	−3.1 (4)
C6—N5—C5—S5	176.84 (17)	C7—N7—C9—S9	177.44 (19)

### Hydrogen-bond geometry (Å, °)

**Table:**

*D*—H···*A*	*D*—H	H···*A*	*D*···*A*	*D*—H···*A*
O1W—H1WA···S3	0.86	2.57	3.284 (2)	140
O1W—H1WB···S9^i^	0.87	2.52	3.355 (2)	161
N2—H2···S4^ii^	0.85 (1)	2.48 (1)	3.3196 (17)	168 (2)
N3—H3···S6^iii^	0.85 (1)	2.48 (1)	3.3203 (17)	168 (2)
N4—H4···S2^ii^	0.86 (1)	2.45 (1)	3.2962 (17)	169 (2)
N5—H5···S1^iii^	0.86 (1)	2.49 (1)	3.3316 (17)	167 (2)
N7—H7···O1W	0.86 (1)	2.03 (1)	2.878 (3)	172 (3)
N9—H9···S8^iv^	0.86 (1)	2.63 (1)	3.470 (2)	167 (3)
N10—H10···N6	0.87 (1)	1.95 (1)	2.804 (3)	168 (3)
N11—H11···N1	0.86 (1)	1.95 (1)	2.795 (2)	164 (3)
N12—H12···S7^v^	0.85 (1)	2.69 (2)	3.439 (2)	148 (3)

Symmetry codes: (i) −*x*, −*y*+2, −*z*; (ii) −*x*+1, −*y*+1, −*z*+1; (iii) −*x*+1, −*y*+1, −*z*; (iv) −*x*, −*y*+2, −*z*+1; (v) *x*, *y*−1, *z*.
